# Facilitators and Barriers Influencing Antipsychotic Medication Prescribing and Deprescribing Practices in Critically Ill Adult Patients: a Qualitative Study

**DOI:** 10.1007/s11606-023-08042-5

**Published:** 2023-04-18

**Authors:** Natalia Jaworska, Karla D. Krewulak, Emma Schalm, Daniel J. Niven, Zahinoor Ismail, Lisa D. Burry, Jeanna Parsons Leigh, Kirsten M. Fiest

**Affiliations:** 1 Department of Critical Care Medicine, University of Calgary, Calgary, AB Canada; 2 Alberta Health Services, Calgary, AB Canada; 3 Department of Community Health Sciences, University of Calgary, Calgary, AB Canada; 4 O’Brien Institute for Public Health, University of Calgary, Calgary, AB Canada; 5 Department of Psychiatry, University of Calgary, Calgary, AB Canada; 6 Hotchkiss Brain Institute, University of Calgary, Calgary, AB Canada; 7 Departments of Pharmacy and Medicine, Sinai Health System, Leslie Dan Faculty of Pharmacy, University of Toronto, Toronto, Canada; 8 School of Health Administration, Faculty of Health, Dalhousie University, Halifax, NS Canada

**Keywords:** antipsychotic medications, prescribing practices, deprescribing, critical care, transitions of care, theoretical domains framework

## Abstract

**Background:**

Antipsychotic medications do not alter the incidence or duration of delirium, but these medications are frequently prescribed and continued at transitions of care in critically ill patients when they may no longer be necessary or appropriate.

**Objective:**

The purpose of this study was to identify and describe relevant domains and constructs that influence antipsychotic medication prescribing and deprescribing practices among physicians, nurses, and pharmacists that care for critically ill adult patients during and following critical illness.

**Design:**

We conducted qualitative semi-structured interviews with critical care and ward healthcare professionals including physicians, nurses, and pharmacists to understand antipsychotic prescribing and deprescribing practices for critically ill adult patients during and following critical illness.

**Participants:**

Twenty-one interviews were conducted with 11 physicians, five nurses, and five pharmacists from predominantly academic centres in Alberta, Canada, between July 6 and October 29, 2021.

**Main Measures:**

We used deductive thematic analysis using the Theoretical Domains Framework (TDF) to identify and describe constructs within relevant domains.

**Key Results:**

Seven TDF domains were identified as relevant from the analysis: *Social/Professional role and identity; Beliefs about capabilities; Reinforcement; Motivations and goals; Memory, attention, and decision processes; Environmental context and resources;* and *Beliefs about consequences*. Participants reported antipsychotic prescribing for multiple indications beyond delirium and agitation including patient and staff safety, sleep management, and environmental factors such as staff availability and workload. Participants identified potential antipsychotic deprescribing strategies to reduce ongoing antipsychotic medication prescriptions for critically ill patients including direct communication tools between prescribers at transitions of care.

**Conclusions:**

Critical care and ward healthcare professionals report several factors influencing established antipsychotic medication prescribing practices. These factors aim to maintain patient and staff safety to facilitate the provision of care to patients with delirium and agitation limiting adherence to current guideline recommendations.

**Supplementary Information:**

The online version contains supplementary material available at 10.1007/s11606-023-08042-5.

## BACKGROUND

Delirium is a common complication of critical illness among adult patients, for which antipsychotic medications are frequently prescribed to manage agitation symptoms.^1–4^ However, large randomized controlled trials have shown that antipsychotic medications do not alter the incidence or duration of delirium in critically ill patients.^5–9^ In 2018, the Society of Critical Care Medicine updated their clinical practice guidelines on the prevention and management of pain, agitation/sedation, delirium, immobility, and sleep disruption recommending against the routine use of antipsychotic medications for delirium.^10^ Adoption of this recommendation remains challenging due to the clinical burden of delirium and agitation, lack of effective alternative pharmacologic interventions to manage agitation symptoms, and lack of clear, delirium-domain targeted approaches for pharmacologic interventions.^6,11,12^ Further, patients prescribed antipsychotic medications in the intensive care unit (ICU) are often discharged from hospital with an ongoing antipsychotic prescription that may no longer be necessary.^13–16^ Utilizing a systematic approach to understand behavioural and socioenvironmental factors influencing antipsychotic prescribing practices may enhance interventions to more effectively and sustainably ensure antipsychotic medications are not unnecessarily continued at hospital discharge.

We utilized the Theoretical Domains Framework (TDF), a 14-domain behaviour change framework, to understand individual and hospital-level factors that influence antipsychotic prescribing practices among healthcare professionals involved in the prescribing and deprescribing of antipsychotic medications in critically ill adults.^17,18^ The objectives of this study were to (1) identify relevant domains that influence antipsychotic prescribing practices among physicians, nurses, and pharmacists that care for critically ill adults during and following critical illness; (2) describe constructs within relevant domains related to antipsychotic prescribing practices; and (3) catalogue potential deprescribing strategies identified by participants for future in-hospital deprescribing initiatives.

## METHODS

### Study Design

This qualitative study is reported according to the Consolidated Criteria for Reporting Qualitative Research checklist (Supplementary Table 1).^19^ Interviews were conducted between July 6 and October 29, 2021. The study was approved by the University of Calgary Conjoint Health Research Ethics Board (REB21-0963).

### Participant Selection

Participants were eligible if they were Alberta physicians, nurses, or pharmacists who spoke English, had a clinical appointment within the hospital environment within the last 5 years, and provided care to critically ill adult patients (i.e., ICU) or patients following critical illness (i.e., on the hospital ward). Participants were recruited using convenience and snowball sampling with eligible participants recruited through social media posts and email invitations. All participants provided informed consent prior to participation.

### Data Collection

Interview guides were developed by the research team and informed by previous interview guides using the TDF within critical care^20^ (Appendix 1–3). Interview guides were piloted with an ICU physician, ICU nurse, and ICU pharmacist prior to their administration. Participants were emailed objectives of the study and a consent form detailing the interview process. One researcher (NJ) trained in qualitative methods conducted all interviews individually with participants over Zoom (Zoom Video Communications, Inc., San Jose, USA). The researcher conducting interviews (NJ) is a critical care physician who had a previous relationship with eight physicians, one nurse, and four pharmacists through clinical work. Interviewer bias was addressed by re-iterating a non-judgemental, confidential environment prior to interview initiation and through the avoidance of leading questions.^21^ The professional relationship of the interviewer provided a collegial and empathizing environment for participants given a commonality of understanding of clinical circumstances and challenges. Interviews ranged from 30 to 60 min. Interviews were audio-recorded for subsequent verbatim transcription. Field notes were made during and after the interview and were revisited between interviews. To ensure credibility, participants were emailed a summary of their interview responses for review, comments, and corrections to ensure their perspectives were accurately interpreted. Nine participants responded with confirmation of accuracy and one participant sent corrections. Twelve participants did not respond to their emailed summary.

### Data Analysis

Verified and de-identified transcripts and interview summary comments were imported into Nvivo12 (QSR International, Melbourne, Australia) for data analysis. Three female researchers (NJ, KDK, ES) trained in deductive qualitative analysis used the TDF framework as the methodologic orientation to complete all data analyses, following the outlined multi-step approach: (1) read text transcripts line-by-line to identify responses and develop a codebook to categorize responses into TDF domains (Supplementary Table 2); (2) develop beliefs from identified responses within their assigned domains; a belief was defined as a collection of responses comprising a similar theme that focused on the problem of ongoing antipsychotic medication prescribing and/or addressed an influence on the target behaviour of antipsychotic deprescribing (20); (3) analyse the beliefs from the domains to identify discrete constructs from the TDF within each domain; and (4) select relevant theoretical domains from the TDF based on their frequency within transcripts, presence of conflicting beliefs, presence of strong beliefs (i.e. longstanding entrenched behaviours) that could impact behaviours, or a combination of all three features.^17,18,20,22^ Relevant domains were determined through discussion between the three researchers (NJ, KDK, ES) using these criteria. Each transcript was coded independently and in duplicate. To address rigour, the researchers utilized cross-examination by having the same transcript reviewed by researchers from different disciplines and backgrounds (e.g. clinicians, researchers). Dependability and confirmability were addressed by maintaining an audit trail and through iterative coding meetings during each data analysis step to clarify coding differences when selecting identified domains. During analysis, the researchers provided reflective commentary to challenge possible conclusions and minimize the risk of bias. Data analysis and data collection occurred in parallel to understand and apply derived codes and constructs to transcripts as new information was garnered. Saturation was achieved across all domains for each healthcare professional role following 21 interviews and was defined by all themes being identified in all healthcare professional role interviews with no new additional beliefs identified. Saturation was not required for all constructs within a domain in order to achieve saturation.

## RESULTS

We completed 21 interviews with 11 physicians, five nurses, and five pharmacists. Thirteen (62%) participants worked primarily in the ICU, and 8 (38%) participants worked primarily on hospital wards. Participants were recruited from six medical centres with 20 (95%) working in an academic environment. Participant characteristics can be found in Table 1. Four participants were recruited via social media (i.e. Twitter) and 12 from the researchers’ personal contacts. Five participants were recruited through snowball sampling from participants forwarding study information to their research networks. All but two participants were actively working within their clinical practice. One ward nurse participant was not actively working within the clinical environment but had been working in their role as a nurse within the last 5 years. One ward pharmacist had retired from clinical practice within the last 6 months at the time they were interviewed.

**Table 1:** Participant Characteristics

Characteristic	Physicians (*n* = 11)	Nurses (*n* = 5)	Pharmacists (*n* = 5)
Age category, years, *n* (%)			
20–29	0 (0%)	2 (40%)	0 (0%)
30–39	7 (64%)	3 (60%)	2 (40%)
40–49	3 (27%)	0 (0%)	2 (40%)
50–59	1 (9%)	0 (0%)	1 (20%)
≥ 60	0 (0%)	0 (0%)	0 (0%)
Sex, *n* (%)			
Female	5 (45%)	5 (100%)	3 (60%)
Work environment, *n* (%)*			
ICU	7 (64%)	3 (60%)	3 (60%)
Ward	4 (36%)	2 (40%)	2 (40%)
Academic	10 (91%)	5 (100%)	5 (100%)
Regional	1 (9%)	0 (0%)	0 (0%)
Work experience in role, years, *n* (%)^†^			
0–5	9 (82%)	3 (60%)	1 (20%)
6–10	1 (9%)	2 (40%)	0 (0%)
≥ 11	1 (9%)	0 (0%)	4 (80%)

^*^Three participants had current or previous roles in both the ICU and ward environment. Work environment was identified as current role in which participants spent ≥ 50% of their clinical time

^†^Two participants were not actively working within clinical practice at the time the interviews were conducted

### Domains Relevant to Antipsychotic Prescribing Practices

All domains were identified from participant interviews. Seven of 14 domains (*n* = 7/14; 50%) and related constructs were relevant to antipsychotic prescribing practices for healthcare professionals (Table 2). Supplementary Table 3 provides a detailed collection of all relevant and non-relevant domains, constructs, and identified beliefs from participants in this study.

**Table 2:** Relevant domains and constructs according to the Theoretical Domains Framework in relation to antipsychotic prescribing practices among critical care and ward healthcare professionals caring for critically ill patients and patients following critical illness

Domains (definition)*	Constructs
Social/professional role and identity *(A coherent set of behaviours and displayed personal qualities of an individual in a social or work setting)*	Professional identity Professional role Professional boundaries Professional confidence Group identity
Beliefs about capabilities *(Acceptance of the truth, reality, or validity about an ability, talent, or facility that a person can put to constructive use)*	Perceived competence Perceived behavioural control Beliefs Empowerment Professional confidence
Reinforcement *(Increasing the probability of a response by arranging a dependent relationship, or contingency, between the response and a given stimulus)*	Incentives Consequents Reinforcement
Motivation and goals *(Mental representations of outcomes or end states that an individual wants to achieve)*	Goal priority Goal/target setting Implementation intention
Memory, attention, and decision processes *(The ability to retain information, focus selectively on aspects of the environment, and choose between two or more alternatives)*	Decision-making Cognitive overload/tiredness
Environmental context and resources *(Any circumstances of a person’s situation or environment that discourage or encourage the development of skills and abilities, independence, social competence, and adaptive behaviour)*	Environmental stressors Resources/material resources Organizational culture/climate Salient events/critical incidents Person × environment interaction Barriers and facilitators
Beliefs about consequences *(Acceptance of the truth, reality, or validity about outcomes of a behaviour in a given situation)*	Beliefs Outcome expectancies Characteristics of outcome Expectancies Anticipated regret Consequents

^*^Definitions of domains from Theoretical Domains Framework developed by Cane et al.[18]

Exemplar quotations for all domains and constructs enumerated below are available in Table 3.

**Table 3:** Exemplar quotations for all identified relevant domains and select constructs within relevant domains

Quotation number (number/participant group)	Constructs	Exemplar quotation
*Social/professional role and identity*		
Quotation number 1 Nurse	Professional identity/boundaries/role	“I think your role as the nurse is to, yeah, to advocate for your patients. So for example, the last time I can think of asking one of my prescribers for Seroquel was, we had a patient who, he was getting a nightly dose of Seroquel to help with his agitation in the ICU, coming out of it. And he was extremely agitated, moving around, crawling out of bed. It was quite unsafe.”
*Beliefs about capabilities*		
Quotation number 2 Pharmacist	Perceived behavioural control	“I try and make a concerted effort to try and stop them before they leave the ICU, if we can, but that’s sometimes not even feasible from within, I would say like 50% of the patients”
*Reinforcement*		
Quotation number 3 Physician	Incentives	“I think one of the things I would think of is what’s the pre-test probability that this patient could cause harm to them or themselves if they’re hyperactive delirium gets worse. So for instance, in a young traumatic brain injury patient who, say he’s a young male who’s strong and is needing four-point restraints, to prevent him from pulling out his ET tube or pulling out his lines, then I think that’s something I would probably lean more towards an early prescription of an antipsychotic to make sure that that doesn’t happen.”
Quotation number 4 Nurse	Reinforcement	“It becomes a point where, if you’re on the floor and you have five other patients and you have a patient that’s requiring one-to-one care because they’re agitated, it’s that kind of thing that you want to ensure that they’re safe.”
*Motivations and goals*		
Quotation number 5 Physician	Goal/target setting	“So I guess the typical prescribing pattern for antipsychotics would be for, probably the three most common ones I would use would be in decreasing frequency, quetiapine or Seroquel, Haldol or olanzapine, and those are typically…or when I use them, I typically use them for either agitated delirium for their antipsychotic benefit, or for either non-specific agitation and sedation, where I tend to use them for their sedating effects.”
Quotation number 6 Nurse	Implementation intervention	“I do value the non-pharmacological interventions over pharmacological interventions. I find myself, I rarely use a PRN antipsychotic if needed. Yeah, I guess on a personal level, I try for the non-pharmacological first”
*Memory, attention, and decision processes*		
Quotation number 7 Nurse	Cognitive overload	“…there are times where you’re saying this is unsafe, and we need to like calm this patient down. But the threshold of what is unsafe is dependent on what else is going on in the unit.”
*Environmental context and resources*		
Quotation number 8 Physician	Organizational culture/climate	“I think, there’s years and years of probably, in my opinion, overprescribing these medications in the intensive care unit. And so then, that builds the culture of, “Well, I’ve seen this for the last 15 or 20 years.” I think that, that’s a big barrier, that kind of institutional inertia.”
Quotation number 9 Pharmacist	Person × environment interactions	“it can be a challenge from time to time, if you have a very busy unit and you have several nurses that are doubled. If they’re doubled with a delirious patient and they’re taking care of their other patients, they might like… Or I don’t want to say like but they might request more pharmacologic management, especially if they’re at risk of pulling out tubes and lines and things, because they still have a second patient to look after and they don’t have time to be right at the bedside monitoring that delirious patient all the time.”
*Beliefs about consequences*		
Quotation number 10 Nurse	Beliefs	“Some people are better at encouraging ‘other’ options for managing patient agitation or sleep than others. I’ve known some really great and creative nurses/HCAs who will play music, sit at patient bedsides, or unit clerks who are happy to keep an eye on patients in chairs, chat with them or while patients colour at the desk or are given a ‘job’ of prepping/ripping labels or ‘folding laundry’, to keep hands busy and out of trouble. These only really work for mild to moderate agitation though, someone who is freaking out doesn’t care about crayons. Orders for “therapeutic touch and massage” or “provide warm milk” come across patronizing and are generally not well received by nursing staff”
Quotation number 11 Physician	Outcome expectancies	“You’re reducing the risk of venous thromboembolic disease, aspiration, pneumonia, falls, fractures, delirium, all the things. Because I think often the antipsychotics just mask the issue as well.”

#### Social/Professional Role and Identity

Healthcare professionals shared their commitment to attempting non-pharmacologic interventions prior to utilizing antipsychotic medications; however, there were differing views regarding utilization of antipsychotic medications when non-pharmacologic management was perceived to be ineffective. In these circumstances, most nurses saw their role as patient advocate. (*Q1*) Pharmacists and physicians more commonly referred to their professional confidence surrounding antipsychotic medication prescription monitoring and safe prescribing practices. One ward physician commented, “we really only prescribe the medications with very specific disclaimers if we are going to use low-dose PRN [*pro re nata*] antipsychotics, and usually we prescribe them kind of with a disclaimer which says something like, ‘This is only to be used for significant agitation or aggression which is putting the individual patient or others at risk of harm.’” All healthcare professionals perceived that antipsychotic prescribing was aligned with their centre’s accepted prescribing practices but highlighted the existence of interdepartmental and individual prescribing differences.

#### Beliefs About Capabilities

 Healthcare professionals across all roles reported holding beliefs around the usefulness of antipsychotics and being the preferred medication due to safer sedation effects. In contrast, some healthcare professionals described their belief of the inefficacy of antipsychotics with one ward nurse commenting on their use for agitation, “I’m not convinced that antipsychotics really help with that in all instances.” Healthcare professionals described their perceived behavioural control defined by variable adherence with known literature on antipsychotic efficacy, reflecting their inconsistent confidence in regulating their own prescribing behaviours. One ICU pharmacist stated, “we’re all aware of some of the conflicting data out there, but I don’t know that they’re [*physicians*] specifically following guidelines every time that they’re prescribing the antipsychotics.” Additionally, healthcare professionals perceived they were participating in antipsychotic deprescribing practices at transitions of care with ward healthcare professionals speaking more directly to their commitment to deprescribing practices than ICU healthcare professionals. (*Q2*) ICU physicians felt professional confidence in their individual antipsychotic prescribing practices and drug administration competency. In contrast, ward physicians described professional confidence in their role of managing deprescribing of antipsychotic medications following patient transfers from the ICU to the ward. ICU nurses but not ward nurses described empowerment in requesting antipsychotic medications as a pharmacologic intervention, particularly during night shifts.

#### Reinforcement

The most reported incentives related to antipsychotics prescribing were patient and staff safety. (*Q3*) This practice was discussed more frequently in the ICU than the hospital ward. As one physician reported, “in the ICU, unless the patient is exhibiting significant agitation that it’s becoming a safety issue, it’s important for me to try and not prescribe an antipsychotic, and especially once a patient’s out of the ICU, my threshold becomes even higher to prescribe an antipsychotic.” Antipsychotic prescribing practices were further reinforced by patient volume and workload (*Q4*) and the perceived variable efficacy of non-pharmacologic interventions. Identified consequents as reinforcements included sedation effects from antipsychotic medications, ease of administering patient care, and the assurance of patient compliance with care.

#### Motivations and Goals

ICU and ward healthcare professionals defined several goal priorities to using antipsychotics including targeting achievement of patient and staff safety, and management of acute hyperactive delirium and agitation. ICU healthcare professionals additionally described weaning sedation, re-establishing day-night routine, and patient comfort as goal priorities. When reflecting on the goals with using antipsychotics, ICU and ward healthcare professionals identified achieving sedation and patient compliance with care delivery as their main goals. (*Q5*) Participants frequently reported attempting to use non-pharmacologic interventions first including family engagement as their primary interventions to prevent and manage delirium. (*Q6*)

#### Memory, Attention, and Decision Processes

Decision-making in both the ICU and hospital ward was influenced by patient-specific factors (e.g., severity of delirium and agitation, patient comorbidities), patient care goals (e.g., quantitative and qualitative sedation targets, patient goals of care), and multidisciplinary team opinions regarding the need for antipsychotics. Cognitive overload was particularly experienced by ward nurses due to high clinical demands and priorities that influenced their recommendations for the use of antipsychotics. (*Q7*)

#### Environmental Context and Resources

ICU healthcare professionals spoke to salient events related to adverse drug effects from antipsychotic prescribing. As one ICU nurse reported, “there was a recent patient who had the serotonin syndrome as a result of antipsychotic use. …and will likely be institutionalized for the rest of his life.” Despite the identification of severe consequences, organizational culture towards antipsychotic use remained unchanged. One nurse compared the ICU and ward culture stating, “in ICU, the general kind of cultural practices, they want calm patients. They’re used to sleeping patients or sedated patients. …whereas on the trauma unit I used to work on, it was pretty normal to have two or three rangy patients that were trying to crawl out of bed for the entire shift.” One physician spoke to the “institutional inertia” within the ICU as culturally driving antipsychotic prescribing practices. (*Q8*) Other organizational culture factors included acceptance of chemical and physical restraints, differences in care goals during night shifts (vs. day shifts), and other unit or healthcare centre prescribing practices. These factors were often reported in the context of available resources, most notably lack of available patient monitoring on the hospital ward and lack of staffing availability in the ICU and on the hospital ward. Environmental stressors that promoted antipsychotic prescribing included unit structure on the ward (e.g. multi-patient rooms, frequent noise) and in the ICU (e.g. lack of windows, lights on at night), patient isolation due to infection protection and control, and the intrusiveness of treatments and care provided in the ICU. Person × environment interactions played a role in antipsychotic prescribing due to patient delirium and agitation severity in both the ICU and on the hospital ward. (*Q9*) Participants highlighted several environmental barriers to minimizing antipsychotic prescribing and engaging in deprescribing including barriers to use of non-pharmacologic management of delirium and agitation (e.g., time constraints), lack of decision-making support tools around antipsychotic prescribing or deprescribing in both the ICU and on the hospital ward, and insufficient communication at transitions of care regarding new medications for ward healthcare professionals. Healthcare professionals in the ICU and on the ward identified family presence and engagement and non-pharmacologic intervention professionals (i.e., geriatricians) as facilitators to antipsychotic deprescribing.

#### Beliefs About Consequences

Participants held multiple beliefs around antipsychotics being important in providing patient and staff safety, and facilitating sleep. Participants in both the ICU and on the ward described contrasting beliefs regarding the efficacy and futility of non-pharmacologic interventions that were dependent on the severity of agitation or delirium. (*Q10*) Few participants viewed that not providing an antipsychotic medication for patients with delirium was a missed delirium treatment opportunity. Characteristics of outcome expectancies identified antipsychotics as being the preferred alternative pharmacologic therapy for delirium with one physician stating, “being afraid to prescribe an antipsychotic might not be the best approach, especially if you’re thinking about alternatives like benzos, which have their own set of side effects and things.” ICU and ward healthcare professionals identified tension between consequents and outcome expectancies. Although they asserted there was a risk of adverse drug effects related to antipsychotic use with additional potential impacts on healthcare system utilization (e.g. increased length of hospital stay, future use of healthcare), participants felt that not using antipsychotics to achieve sedation in patients with delirium or agitation exposed patients to delays in therapy delivery (e.g., mobilization), could add additional healthcare system utilization costs, and could cause family distress. Other healthcare professionals reported the contrary that antipsychotics were responsible for delays in therapy (e.g., identification of new clinical issues) and lack of participation in care. (*Q11*) Antipsychotic use was associated with anticipated regret around adverse effects, lack of deprescribing practices, and ongoing unnecessary antipsychotic prescriptions.

### Participant-Identified Suggested Deprescribing Strategies

Participants shared suggestions of antipsychotic deprescribing strategies to reduce the proportion of critically ill patients discharged from hospital with an ongoing unnecessary antipsychotic prescription. Most participants (*n* = 15, 71%) suggested the use of a direct communication tool (e.g., embedded deprescribing recommendations in transfer/discharge summaries, direct standardized verbal communication regarding deprescribing with new accepting care providers) between prescribers at transitions of care in addition to commonly completed transfer summaries. For example, participants discussed inclusion of specific instructions within transfer summaries to identify high-risk medications, such as antipsychotics, and provide explicit instructions on discontinuation recommendations. Additional recommendations by participants (*n* = 15, 71%) focused on antipsychotic prescribing accountability practices including force-function alerts to identify antipsychotics for review, and automatic stop dates (Table 4).

**Table 4:** Participant-Identified Deprescribing Strategies

Deprescribing strategy	Participants reporting on strategy (%)*
Direct communication tool between prescribers at transitions of care (e.g., embedded deprescribing recommendations in transfer/discharge summaries, direct standardized verbal communication on deprescribing with new accepting care providers)	15 (71%)
Antipsychotic medication prescribing accountability (e.g., automatic stop dates, no as-needed dosing)	15 (71%)
Additional medication reconciliation at transitions of care	10 (48%)
Formal education sessions on indications for antipsychotic medication prescribing and deprescribing	9 (43%)
Expert consultations on medication management upon transition of care (e.g., geriatrics consultation, outpatient follow-up)	4 (19%)
Pharmacist-driven deprescribing strategies or algorithms	4 (19%)
Tapering protocols and discharge medication care bundles	4 (19%)
Antipsychotic prescribing policy development	1 (5%)
Practice audits	1 (5%)

^*^Percentages do not add up to 100 due to the possibility of multiple reported strategies per participant

## DISCUSSION

In this multi-centre, qualitative study of 21 critical care and hospital ward physicians, nurses, and pharmacists, seven relevant TDF domains and their associated constructs were identified as impacting antipsychotic medication prescribing practices of healthcare professionals for adult patients with and following critical illness. These domains included *Social/Professional role and identity; Beliefs about capabilities; Reinforcement; Motivations and goals; Memory, attention, and decision processes; Environmental context and resources;* and *Beliefs about consequences*. Participant-generated recommendations further identified antipsychotic deprescribing strategies to facilitate safe in-hospital practice pattern changes through direct communication tools between prescribers at transitions of care and strategies to ensure antipsychotic medication prescribing accountability. Although important potential deprescribing recommendations were identified by participants, these strategies remain exploratory and do not represent an exhaustive list of potential interventions.

Our data further suggest that antipsychotic medications are being prescribed for multiple indications besides delirium such as patient and staff safety, sleep management, and to manage environmental factors such as staff availability and workload. Individual and group beliefs as well as organizational structures, processes, and resource constraints appear to play an important role in why antipsychotic medications are prescribed and continued throughout a critically ill patient’s hospitalization and may at least partially explain the disconnect between guideline recommendations and current antipsychotic prescribing practices. Our study highlights the lack of structured antipsychotic prescribing guidelines and deprescribing assessments at all transitions of care. These handovers of patient care may benefit from our identified antipsychotic deprescribing strategies as interventions to ensure antipsychotic medications are not continued inappropriately in patients who experience critical illness.

Few studies have attempted to implement antipsychotic deprescribing interventions among critically ill patients at transitions of care.^23–25^ These studies have had variable success in effectively and sustainably reducing the number of critically ill patients discharged from hospital with ongoing antipsychotic prescriptions by utilizing education, hand-off tools and algorithms, and pharmacist-driven prescriptive deprescribing authority as interventions.^23–25^ This may be due to a lack of evidence-based rationale for implementation strategies addressing factors influencing healthcare professional behaviours. The relevant domains from the TDF related to antipsychotic prescribing practices identified in our study provide an understanding of prescribing behaviours and what behaviours may need to be addressed to facilitate antipsychotic deprescribing. Our results provide the outline for application of these domains to additional knowledge translation frameworks such as the Capability, Opportunity, Motivation, Behaviour (COM-B) model and the Behaviour Change Wheel to develop interventions and policy strategies around antipsychotic prescribing practices.^26^ For example, several identified relevant domains (i.e., *Beliefs about consequences, Beliefs about capabilities, Motivations and goals, Social/professional role and identity*) suggest that addressing prescriber motivation may be effective in mitigating ongoing antipsychotic prescriptions at transitions of care. These interventions and policy strategies may include additional training related to medication reconciliation and deprescribing at transitions of care and additional support with medication reconciliation through authoritative prescribing capabilities of pharmacists to implement antipsychotic deprescribing at transitions of care. Further knowledge translation interventions to modify or reduce ongoing antipsychotic prescriptions may also include environmental restructuring (e.g., policy changes related to antipsychotic orders) through antipsychotic prescribing and deprescribing guidelines and regulations, as well as the utilization of incentivization techniques (e.g., public recognition or awards for improvements in deprescribing). Additionally, feedback on prescribing behaviours may also effectively impact antipsychotic prescribing and deprescribing behaviours.^27^

Our study has several strengths including the recruitment of a broad sample of multidisciplinary healthcare professionals providing a comprehensive understanding of antipsychotic prescribing practices across transitions of care for critically ill patients. Additionally, our results have theoretical generalizability given the use of a previously validated framework.^18^ Saturation was achieved for all domains but not constructs for each healthcare professional role. Not all constructs reported were identified by all healthcare professional roles as certain perceptions were likely discipline specific. There may be discipline-specific barriers that could be addressed; however, this was not the aim of the current study. When looking to identify strategies to facilitate behaviour change related to antipsychotic prescribing practices, system-based strategies are needed as the actions of healthcare professionals are interconnected so that behaviour changes for one discipline change the context for all other disciplines.^28,29^ Due to pandemic constraints, we used convenience and snowball sampling for participant recruitment with most participants working in academic medical centres. It is possible that this sampling technique may have missed perspectives from healthcare professionals in other hospital structures (i.e., regional or community) that may have important and unique perspectives related to antipsychotic medication prescribing. Additionally, our participants represent a relatively young sample across all healthcare professional roles and may not have identified relevant themes from healthcare professionals with more years of clinical experience. Further, all interviews were completed by one interviewer who had professional relationships with 13 participants which despite best efforts to create a non-judgmental confidential environment (e.g., interviews completed individually, empathetic listening) may have introduced bias in the participant interview responses limiting what they were comfortable sharing. Interviews took place during the COVID-19 pandemic which may have impacted some of the responses offered by participants. Lastly, the use of the TDF precluded the inclusion of patient and family/caregiver perspectives which was beyond the scope of this study. Future studies should ensure that perspectives from these important stakeholder groups are included.

## CONCLUSIONS

Critical care and ward healthcare professionals report their antipsychotic prescribing practices being rooted in maintaining patient and staff safety while delivering appropriate clinical care in patients with delirium and agitation. Although well-intentioned, antipsychotic prescribing in critical care is haphazard and not guideline based. Future interventions to reduce antipsychotic prescribing and promote antipsychotic deprescribing at transitions of care should address the identified relevant domains.

## Supplementary Information

Below is the link to the electronic supplementary material.

Supplementary file1 (DOCX 51 KB)

## Data Availability

All data are available in published records.

## Data Availability

Data generated during and/or analysed during the current study are available from the corresponding author on reasonable request.
