# Supplementary material for: Facilitators and Barriers Influencing Antipsychotic Medication Prescribing and Deprescribing Practices in Critically Ill Adult Patients: a Qualitative Study
Source: J Gen Intern Med. 2023 Apr 18;38(10):2262–71. doi: 10.1007/s11606-023-08042-5 (PMC10112822; doi:10.1007/s11606-023-08042-5)
Supplement: Supplementary file 1 — Supplementary file1 (DOCX 51 KB) [file 11606_2023_8042_MOESM1_ESM.docx]

**Appendices**

**Supplementary Table 1.** Consolidated criteria for Reporting Qualitative research (COREQ) Checklist.

| **Domain 1: Research team and reﬂexivity** | | |
| --- | --- | --- |
| *Personal Characteristics* | | *Location in Manuscript, section (page number)* |
| Which author/s conducted the interview or focus group? | NJ | Title page |
| What were the researcher’s credentials? E.g. PhD, MD | NJ (MD, MSc), KDK (PhD), ES (BKin/BCom), DJN (MD, MSc, PhD), ZI (MD), LDB (PharmD), JPL (PhD), KMF (PhD) | Title Page |
| What was their occupation at the time of the study? | NJ (Attending Physician), KDK (Research Associate), ES (Graduate Student), DJN (Attending Physician, Assistant Professor), ZI (Attending Physician, Associate Professor), LDB (Pharmacist, Assistant Professor), JPL (Assistant Professor), KMF (Associate Professor) | Not reported in manuscript |
| Was the researcher male or female? | Female: NJ, KDK, LDB, JPL, KMF  Male: DJN, ZI | Methods (page 6) |
| What experience or training did the researcher have? | All (training in qualitative methods, facilitator experience) | Methods (page 5-6) |
| *Relationship with participants* | | |
| Was a relationship established prior to study commencement? | Yes | Methods (Page 5) |
| What did the participants know about the researcher? e.g. personal goals, reasons for doing the research | Participants received an email outlining the objectives of the study, verification of ethical approval, and an informed consent form detailing the interview process. An additional oral consent process was completed with all participants and an opportunity to ask and answer all questions occurred prior to commencement of the semi-structured interview. | Methods (Pages 5-6) |
| What characteristics were reported about the interviewer/facilitator? e.g., Bias, assumptions, reasons and interests in the research topic | Interviewer bias, participants aware of interviewer’s interest in research topic | Methods (Pages 5-7) |
| **Domain 2: Study design** | | |
| *Theoretical framework* | | |
| What methodological orientation was stated to underpin the study? e.g. grounded theory, discourse analysis, ethnography, phenomenology, content analysis | Deductive thematic analysis using the Theoretical Domains Framework | Methods (Page 6) |
| *Participant Selection* | | |
| How were participants selected? e.g. purposive, convenience, consecutive, snowball | Convenience and snowball | Methods (Page 5) |
| How were participants approached? e.g. face-to-face, telephone, mail, email | Recruited via e-mail, social media | Methods (Page 5) |
| How many participants were in the study? | 21 | Results (Page 7) |
| How many people refused to participate or dropped out? Reasons? | Not applicable | Not applicable |
| *Setting* | | |
| Where was the data collected? e.g. home, clinic, workplace | Virtually via Zoom | Methods (Page 5) |
| Was anyone else present besides the participants and researchers? | No | Methods (Page 5) |
| What are the important characteristics of the sample? e.g. demographic data, date | Demographic data | Table 1, Results |
| *Data collection* | | |
| Were questions, prompts, guides provided by the authors? Was it pilot tested? | Interview guides were provided to participants by the authors. All interview guides were pilot tested with an ICU RN, ICU physician, and ICU pharmacist. | Methods (Page 5), Appendix 1-3 |
| Were repeat interviews carried out? If yes, how many? | No | Not reported in manuscript |
| Did the research use audio or visual recording to collect the data? | All semi-structured interviews were audio-recorded only using the audio recording feature on Zoom; participants were asked to turn off their cameras for the duration of the interview | Methods (Page 6) |
| Were field notes made during and/or after the interview or focus group? | Yes, but field notes were not utilized in the data analysis | Methods (Page 6) |
| What was the duration of the interviews or focus group? | All interviews lasted approximately 30 minutes to 1 hour | Methods (Page 6) |
| Was data saturation discussed? | Yes | Methods (Page 7) |
| Were transcripts returned to participants for comment and/or correction? | No. A personalized summary of the interview was sent to participants to review and provide comments and/or corrections | Methods (Page 6) |
| **Domain 3: analysis and findings** | | |
| *Data analysis* | | |
| How many data coders coded the data? | Three (NJ, KDK, ES) | Methods (Page 6) |
| Did authors provide a description of the coding tree? | Yes | Methods (page 6, 7) |
| Were themes identified in advance or derived from the data? | Themes were identified in advance using the Theoretical Domains Framework | Methods (Page 6) |
| What software, if applicable, was used to manage the data? | NVivo12 | Methods (Page 6) |
| Did participants provide feedback on the findings? | A summary of the interview was returned to the participants for additional comments and/or corrections | Methods (Page 6) |
| *Reporting* | | |
| Were participant quotations presented to illustrate the themes/findings? Was each quotation identified? e.g., participant number | Yes. Quotations were presented by participant role | Results (Pages 7-14), Table 3 |
| Was there consistency between the data presented and the findings? | Yes | Results (Pages 7-14), Table 3 and Supplementary Table 3 |
| Were major themes clearly presented in the findings? | Yes | Results (Pages 7-14), Table 2, Table 3 and Supplementary Table 3 |
| Is there a description of diverse cases or discussion of minor themes? | Yes | Results (Page 7), Supplementary Table 3 |

**Supplementary Table 2.** The Theoretical Domains Framework v2 developed by Cane *et al.*[18] used as a methodologic orientation for data analysis

| Domains (definition) | Constructs |
| --- | --- |
| Knowledge  *(an awareness of the existence of something)* | Knowledge (including knowledge  of condition/scientific rationale)  Procedural knowledge  Knowledge of task environment |
| Skills  *(an ability or proficiency acquired through practice)* | Skills  Skills development  Competence  Ability  Interpersonal skills  Practice  Skill assessment |
| Social/professional role and identity  *(a coherent set of behaviours and displayed personal qualities of an individual in a social or work setting)* | Professional identity (i.e., characteristics by which an individual is recognized to relate or connect to a particular profession)  Professional role (i.e., behaviour considered appropriate for a particular kind of work)  Social identity  Identity  Professional boundaries  Professional confidence (i.e., belief in one’s repertoire of skills)  Group identity  Leadership  Organizational commitment |
| Beliefs about capabilities  *(acceptance of the truth, reality or validity about an ability, talent or facility that a person can put to constructive use)* | Self-confidence  Perceived competence (i.e., belief in ability to learn or execute skills)  Self-efficacy  Perceived behavioural control (i.e., perception of ease/difficulty of performing behaviour)  Beliefs  Self-esteem  Empowerment  Professional confidence (i.e., belief in one’s repertoire of skills) |
| Optimism  *(the confidence that things will happen for the best or that desired goals will be attained)* | Optimism  Pessimism  Unrealistic optimism (i.e., tendency to over-rate individual abilities and chance of positive outcome compared to others)  Identity |
| Reinforcement  *(increasing the probability of a response by arranging a dependent relationship, or contingency, between the response and a given stimulus)* | Rewards (proximal/distal, valued/not valued, probable/improbable)  Incentives  Punishment  Consequents (i.e., outcome of behaviour in given situation)  Reinforcement  Contingencies (i.e., conditional probabilistic relation between two events)  Sanctions |
| Intentions  *(a conscious decision to perform a behaviour or a resolve to act in a certain way)* | Stability of intentions (i.e., ability of individual resolve to remain in spite of disturbing influences)  Stages of change model (i.e., model of five stage behaviour change: pre-contemplation, contemplation, preparation, action, maintenance)  Transtheoretical model and stages of change (i.e., five-stage theory of health behaviour change: change takes time and different interventions effective at different stages) |
| Motivation & goals  *(mental representations of outcomes or end states that an individual wants to achieve)* | Goals (distal/proximal) (i.e., desired state of affairs of person or system which may be closer (proximal) or further away (distal))  Goal priority  Goal/target setting  Goals (autonomous/controlled) (i.e., end state towards which individual is striving; purpose of an activity)  Action planning  Implementation intention (i.e., plan individual creates to enact a behaviour) |
| Memory, attention & decision processes  *(the ability to retain information, focus selectively on aspects of the environment and choose between two or more alternatives)* | Memory  Attention  Attention control  Decision making  Cognitive overload/tiredness |
| Environmental context & resources  *(any circumstances of a person’s situation or environment that discourages or encourages the development of skills and abilities, independence, social competence and adaptive behaviour)* | Environmental stressors  Resources/material resources  Organizational culture/climate  Salient events/critical incidents  Person x environment interaction (i.e., interplay between individual and their surroundings)  Barriers and facilitators |
| Social influences  *(those interpersonal processes that can cause individuals to change their thoughts, feelings or behaviours)* | Social pressure  Social norms  Group conformity  Social comparisons  Group norms  Social support  Power  Intergroup conflict  Alienation  Group identity  Modelling |
| Emotion  *(a complex reaction pattern, involving experiential, behavioural, and physiological elements, by which the individual attempts to deal with a personally significant matter or event)* | Fear  Anxiety  Affect  Stress  Depression  Positive/negative affect  Burn-out |
| Behavioural regulation  *(anything aimed at managing or changing objectively observed or measured actions)* | Self-monitoring  Breaking habit  Action planning |
| Beliefs about consequences  *(acceptance of the truth, reality, or validity about outcomes of a behaviour in a given situation)* | Beliefs  Outcome expectancies (i.e., cognitive, emotional, behavioural outcomes assumed to be associated with future behaviours)  Characteristics of outcome expectancies (i.e., characteristics of cognitive, emotional, behavioural outcomes individuals believe are associated with future behaviours)  Anticipated regret (i.e., sense of potential negative consequences that influence decisions made)  Consequents (i.e., outcome of behaviour in given situation) |

**Supplementary Table 3.** Relevant and non-relevant domains, constructs, and beliefs related to antipsychotic prescribing practices among critical care and ward healthcare professionals caring for critically ill patients and patients following critical illness

| **Relevant Domains** | | |
| --- | --- | --- |
| ***Domains*** | ***Constructs*** | ***Beliefs*** |
| Social/professional role & identity | Professional confidence | - Prescription monitoring and safe prescribing practices (e.g., deprescribing ICU orders, prescribing for appropriate indications)  - Self-identified knowledge gaps (e.g., know more about antipsychotics, knowledge about the patient) |
|  | Professional identity/boundaries/role | - Perceived role in implementation of non-pharmacologic interventions  - Patient advocacy  - Healthcare professional boundaries between multidisciplinary team members |
|  | Group identity | - Accepted prescribing practices  - Collaboration in multidisciplinary environment  - Interdepartmental and healthcare professional prescribing differences |
| Beliefs about capabilities | Professional competence | - Implementation of non-pharmacologic and pharmacologic interventions  - Perceived deprescribing practices |
|  | Perceived behavioural control | - Guideline adherence  - Differential confidence in implementing prescribing recommendations |
|  | Professional confidence | - Drug administration competency and appropriate monitoring  - Antipsychotic management at transition of care  - Night shift pharmacologic management |
|  | Empowerment | - Individualized healthcare professional prescribing practices  - Nurse requests for pharmacologic interventions |
|  | Beliefs | - Acceptance of truth that antipsychotics useful for hyperactive delirium and agitation  - Preferred safe sedation effects  - Inefficacy of antipsychotics |
| Reinforcement | Consequents | - Sedation effects  - Ease of patient care and patient compliance with care (eg. Less work, on-call calls) |
|  | Incentives | - Patient and staff safety  - Ease of pharmacologic administration  - Agitation and delirium as routine event |
|  | Reinforcements | - Patient volume and workload  - Implementation and efficacy of non-pharmacologic interventions (e.g., failure of non-pharmacologic management in hyperactive delirium) |
| Motivation & goals | Goal priority | - Weaning sedation  - Day-night routine  - Patient and staff safety  - Acute hyperactive delirium and agitation management  - Comfort |
|  | Goal/target setting | - Sedation  - Patient compliance |
|  | Implementation intervention | - Attempt to use non-pharmacologic interventions first  - Family engagement |
| Memory, attention & decision processes | Decision making | - Patient specific factors (eg. QTc, age, comorbidities, severity of delirium and agitation)  - Multidisciplinary team opinions  - Patient care goals (eg. RASS, quantitative and qualitative assessments, weaning sedation)  - Timing of deprescribing practices |
|  | Cognitive overload | - Other clinical pressures and priorities |
| Environmental context & resources | Salient event/critical incidents | - Adverse effect events (e.g., severe long-term disability, death) |
|  | Environmental stressors | - Unit specific considerations (e.g., sedation and agitation thresholds)  - Unit physical structure (e.g., windows, multi-patient rooms, lights, noise)  - Patient isolation  - Intrusiveness of treatments provided in the ICU |
|  | Resources/material resources | - Staffing availability  - Availability of monitoring |
|  | Organizational culture/climate | - Workplace culture around antipsychotic prescribing threshold  - Chemical and physical restraints  - Institutional inertia  - Day vs night shift differences  - Other healthcare centre and unit prescribing practices |
|  | Person x environment interactions | - Patient environment interactions (i.e., delirium and agitation) |
|  | Barriers & facilitators | - Barriers to non-pharmacologic management of delirium and agitation  - Lack of decision-making support and policy/protocols  - Communications at transitions of care |
| Beliefs about consequences | Outcome expectancies | - Sedation effects of antipsychotics  - Delay in therapy delivery (e.g., diagnostics, mobilization)  - Family distress |
|  | Characteristics of outcome expectancies | - Antipsychotics preferred alternative therapy |
|  | Anticipated regret | - Lack of deprescribing practices  - Ongoing antipsychotic prescriptions without clinical indication  - Adverse medication effects (e.g., oversedation) |
|  | Consequents | - Risk of adverse effects  - Impacts on healthcare system utilization (e.g., future use of healthcare, length of stay, increased cost) |
|  | Beliefs | - Perception that patients should sleep at night  - Effectiveness vs. futility of non-pharmacologic interventions, antipsychotics, and deprescribing practices  - Patient and staff safety  - Missed delirium treatment opportunity when not using antipsychotics |
| **Non-relevant Domains** | | |
| ***Domains*** | ***Constructs*** | ***Beliefs*** |
| Knowledge | Knowledge about condition/scientific rationale | - Awareness of guidelines  - Knowledge of pharmacologic effects (e.g., randomized control trials)  - Knowledge of delirium bundle and non-pharmacologic interventions |
| Skills | Skills | - Applied knowledge (e.g., clinical experience, ability to identify etiologies of delirium)  - Consistent identification of delirium and agitation |
|  | Skill development | - Formal skill development (e.g., basic pharmacologic knowledge of drugs)  - Informal skill development (e.g., clinical training programmes) |
|  | Ability | - Communication (i.e., between healthcare professionals and patients/families) |
| Optimism | Optimism | - Slow change to prescribing practices  - Differential optimism (e.g., context dependent, optimism in deprescribing benefit)  - Poor alternative therapeutic options |
| Intentions | Stability of intentions | - Influence of workplace and unit culture  - Perceived responsible prescribing practices  - Perceived deprescribing and de-intensifying practices at transition of care |
|  | Stages of change model | - Awareness and use of delirium bundle  - Pre-contemplation and indifference in antipsychotic prescribing behaviour regulation |
| Social influences | Social support | - Multidisciplinary approach to antipsychotic prescribing decisions |
|  | Group identity | - Hierarchy of social influence between healthcare professionals  - Belief in other healthcare professional knowledge  - Family influence |
|  | Group conformity | - Ingrained prescribing practices  - Negotiations between healthcare professionals around antipsychotic prescribing  - Multidisciplinary rounds and conversations to determine antipsychotic prescribing |
|  | Social comparisons | - Individual prescriber practice patterns  - Differential perceptions of antipsychotic efficacy between different healthcare groups (e.g., ICU vs. ward physicians) |
|  | Group norms | - Professional boundaries and roles |
| Emotion | Burn-out | - Emotional and physical exhaustion with delirious and agitated patients |
|  | Fear | - Worry about patient and staff safety |
|  | Anxiety | - Anticipatory anxiety about availability of antipsychotics  - Concern about ongoing antipsychotic prescribing at transitions of care |
|  | Stress | - Frustration about limited available effective diagnostics and treatments for delirium  - Frustration with multiple opinions and questions surrounding antipsychotic prescribing requests |
|  | Affect | - Sense of futility with delirium management  - Hopelessness around identifying effective treatment other than antipsychotics to treat delirium and agitation  - Confusion (e.g., delirium research nebulous and changing frequently) |
| Behavioural regulation | Self-monitoring | - Antipsychotic de-escalation prescribing practices  - Individual prescriber practices to regulate antipsychotic prescribing (e.g., review need for medications daily)  - Intention to regulate antipsychotic prescribing behaviour |
|  | Action planning | - Delirium discussion at rounds |

**Appendix 1.** Physician interview guide


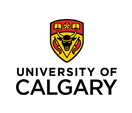


**Facilitators and barriers to deprescribing antipsychotic medications in critically ill adult patients at transitions of care: A mixed methods study**

**Interview Guide**

Introduction:

Thank you for agreeing to speak with me today about antipsychotic medication prescribing and deprescribing in patients with critical illness and following critical illness. We are conducting interviews with ICU and ward physicians, nurses and pharmacists across Alberta. We look forward to hearing about your experiences and expert knowledge. These topics serve as a guide only. If there are other details that you would like to share, I would like to hear them.

You were emailed a copy of the informed consent form. The consent form is part of the process of informed consent. It should give you an idea of what this research is about and your role as a participant.

Did you receive the consent form and have a chance to read it?

Because it is important that you understand your rights as a participant, we will review the main components of the consent form here.

[Read/review oral consent form]

Before we start, I would like to remind you that we will be audio recording this interview so that we can accurately capture our conversation. Do you agree to be audio recorded for research purposes?

I will start audio recording now. [Start audio recording]

Can you please verbally state that you consent to participate in this study?

Antipsychotic medications such as quetiapine and haloperidol are commonly prescribed to adult patients in the intensive care unit with non-psychiatric diagnoses. These medications are frequently continued at transitions of care in critically ill adult patients. Today we will be talking about your experience with prescribing and deprescribing antipsychotic medications in critically ill patients while in the ICU, following their transfer to the ward, and to hospital discharge. In 2018, the Society of Critical Care Medicine published clinical practice guidelines for the prevention and management of pain, agitation/sedation, delirium, immobility, and sleep disruption in adult patients in the ICU.

**Knowledge**

1. Are you aware of current clinical practice guidelines for antipsychotic medication use in critically ill patients?
2. What is your understanding of clinical practice guidelines for antipsychotic medication use in critically ill patients?
3. Do you or does your unit use any parts of this guideline when prescribing antipsychotic medications to patients in the ICU or those patients transferred from the ICU?

**Social/Professional role and identity**

1. Does your role as a physician influence how you prescribe antipsychotic medications for critically ill patients or those patients that have experienced critical illness? How so?

(Prompt: For example, have you previously been trained to prescribe antipsychotics? Is this standard of practice? Is this an accepted prescribing practice in your unit?)

**Social influences**

1. Are there other healthcare providers that would influence whether or not you prescribe antipsychotics to critically ill patients or those patients who have been critically ill?

(Prompt: If so, who would that be and in what circumstance would that influence your behaviours?)

**Behavioural regulation**

1. Are there any policies or procedures in place that provide guidance on antipsychotic medication prescribing in the ICU or on the ward?
2. If you were going to not prescribe an antipsychotic medication, how confident would you be that this could be carried out in your unit?

**Skills**

1. What skills or skill set are required for you to make a decision to prescribe an antipsychotic medication to a critically ill patient or a patient that has been recently critically ill?

**Beliefs about capabilities**

1. What problems or challenges might you expect to encounter if you were to manage a patient without an antipsychotic medication?
2. What aspects of patient care or the professional provision of care might help to overcome these problems or challenges?

**Environmental context and resources**

1. Are there ways that the clinical environment either in the ICU or on the ward affects the use of antipsychotic medications?

(Prompt: If so, how does the clinical environment affect the use of antipsychotic medications?)

**Beliefs about consequences**

1. What are the benefits of not prescribing a critically ill patient or a patient that has recently been critically ill antipsychotic medications?

(Prompts: to yourself, to the patient, to the healthcare system?)

1. What disadvantages are there in not prescribing antipsychotic medications to a critically ill patient or a patient that has recently been critically ill?

(Prompts: to yourself, to the patient, to the healthcare system?)

1. Are there incentives in the ICU or on the ward to not prescribe patients antipsychotic medications?

**Motivations and goals**

1. How important is it to you that you do not prescribe antipsychotic medications to critically ill patients or those who have recently been critically ill?

**Memory, attention and decision processes**

1. What factors might play a role in your decision-making process when deciding to prescribe antipsychotic medications to critically ill patients or those patients who have been recently critically ill?
2. In what situations would you find it difficult to use alternative interventions (ie. non-pharmacologic or pharmacologic) other than antipsychotic medications in managing critically ill patients or those who have been recently critically ill?

**Emotion**

1. Are there any situations that you would have feelings of worry about not prescribing an antipsychotic medication?

**Intentions**

1. To what extent in your daily clinical practice do you intentionally regulate your prescribing of antipsychotic medications?

**Optimism**

1. How confident are you that reducing antipsychotic prescribing will improve patient care in the future?

(Prompt: Are you optimistic, pessimistic, or indifferent?)

**Reinforcement**

1. Are there any rewards or consequences if antipsychotic medications are prescribed to critically ill patients or those patients who have recently been critically ill?

(Prompts: to yourself? To patients? To the healthcare system?)

**Closing questions**

1. What potential strategies or approaches could be used to prevent critically ill patients from being discharged from hospital with antipsychotic medications that do not have a clinical indication?
2. Do you have anything you would like to share on this topic that we haven’t discussed today?

Thank you for participating in our study. I will stop recording now and will ask some demographic questions.

[Stop recording]

**Appendix 2.** Pharmacist and nursing interview guide


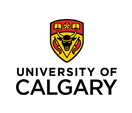


**Facilitators and barriers to deprescribing antipsychotic medications in critically ill adult patients at transitions of care: A mixed methods study**

**Interview Guide**

Introduction:

Thank you for agreeing to speak with me today about antipsychotic medication prescribing and deprescribing in patients with critical illness and following critical illness. We are conducting interviews with ICU and ward physicians, nurses and pharmacists across Alberta. We look forward to hearing about your experiences and expert knowledge. These topics serve as a guide only. If there are other details that you would like to share, I would like to hear them.

You were emailed a copy of the informed consent form. The consent form is part of the process of informed consent. It should give you an idea of what this research is about and your role as a participant.

Did you receive the consent form and have a chance to read it?

Because it is important that you understand your rights as a participant, we will review the main components of the consent form here.

[Read/review oral consent form]

Before we start, I would like to remind you that we will be audio recording this interview so that we can accurately capture our conversation. Do you agree to be audio recorded for research purposes?

I will start audio recording now. [Start audio recording]

Can you please verbally state that you consent to participate in this study?

Antipsychotic medications such as quetiapine and haloperidol are commonly prescribed to adult patients in the intensive care unit with non-psychiatric diagnoses. These medications are frequently continued at transitions of care in critically ill adult patients. Today we will be talking about your experience with prescribing and deprescribing antipsychotic medications in critically ill patients while in the ICU, following their transfer to the ward, and to hospital discharge. In 2018, the Society of Critical Care Medicine published clinical practice guidelines for the prevention and management of pain, agitation/sedation, delirium, immobility, and sleep disruption in adult patients in the ICU.

**Knowledge**

1. Are you aware of current clinical practice guidelines for antipsychotic medication use in critically ill patients?
2. What is your understanding of clinical practice guidelines for antipsychotic medication use in critically ill patients?
3. Do you or does your unit use any parts of this guideline when prescribing antipsychotic medications to patients in the ICU or those patients transferred from the ICU?

**Social/Professional role and identity**

1. Does your role as a [pharmacist/nurse] influence how you recommend prescribing antipsychotic medications for critically ill patients or those patients that have experienced critical illness? How so?

(Prompt: For example, have you previously been trained to recommend prescribing antipsychotics? Is this standard of practice? Is this an accepted prescribing practice in your unit?)

**Social influences**

1. Are there other healthcare providers that would influence whether or not you recommend prescribing antipsychotics to critically ill patients or those patients who have been critically ill?

(Prompt: If so, who would that be and in what circumstance would that influence your behaviours?)

**Behavioural regulation**

1. Are there any policies or procedures in place that provide guidance on antipsychotic medication prescribing in the ICU or on the ward?
2. If you were going to recommend against prescribing an antipsychotic medication, how confident would you be that this could be carried out in your unit?

**Skills**

1. What skills or skill set are required for you to make a decision to recommend prescribing an antipsychotic medication to a critically ill patient or a patient that has been recently critically ill?

**Beliefs about capabilities**

1. What problems or challenges might you expect to encounter if you were to manage a patient without an antipsychotic medication?
2. What aspects of patient care or the professional provision of care might help to overcome these problems or challenges?

**Environmental context and resources**

1. Are there ways that the clinical environment either in the ICU or on the ward affects the use of antipsychotic medications?

(Prompt: If so, how does the clinical environment affect the use of antipsychotic medications?)

**Beliefs about consequences**

1. What are the benefits of not recommending prescribing a critically ill patient or a patient that has recently been critically ill antipsychotics medications?

(Prompts: to yourself, to the patient, to the healthcare system?)

1. What disadvantages are there in not recommending prescribing antipsychotic medications to a critically ill patient or a patient that has recently been critically ill?

(Prompts: to yourself, to the patient, to the healthcare system?)

1. Are there incentives in the ICU or on the ward to not recommend prescribing patients antipsychotic medications?

**Motivations and goals**

1. How important is it to you that you recommend against prescribing antipsychotic medications to critically ill patients or those who have recently been critically ill?

**Memory, attention and decision processes**

1. What factors might play a role in your decision-making process when deciding to recommend prescribing antipsychotic medications to critically ill patients or those patients who have been recently critically ill?
2. In what situations would you find it difficult to use alternative interventions (ie. non-pharmacologic or pharmacologic) other than antipsychotic medications in managing critically ill patients or those who have been recently critically ill?

**Emotion**

1. Are there any situations that you would have feelings of worry about not recommending the prescribing of an antipsychotic medication?

**Intentions**

1. To what extent in your daily clinical practice do you intentionally regulate your recommendations of prescribing antipsychotic medications?

**Optimism**

1. How confident are you that reducing antipsychotic prescribing will improve patient care in the future?

(Prompt: Are you optimistic, pessimistic, or indifferent?)

**Reinforcement**

1. Are there any rewards or consequences if antipsychotic medications are prescribed to critically ill patients or those patients who have recently been critically ill?

(Prompts: to yourself? To patients? To the healthcare system?)

**Closing questions**

1. What potential strategies or approaches could be used to prevent critically ill patients from being discharged from hospital with antipsychotic medications that do not have a clinical indication?
2. Do you have anything you would like to share on this topic that we haven’t discussed today?

Thank you for participating in our study. I will stop recording now and will ask some demographic questions.

[Stop recording]

**Appendix 3. Collected participant demographics guide.**


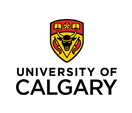


**Facilitators and barriers to deprescribing antipsychotic medications in critically ill adult patients at transitions of care: A mixed methods study**

**Post-interview demographic survey**

**Demographic Questions**

We are collecting personal demographic information to describe our participants in aggregate. Any contact information you provide us will only be used to share a summary of our conversation here today if you would like to review it to ensure it reflects your thoughts. Please note that your demographic information and contact info will be stored in a password protected database that is only accessible to the study research team. If you are not comfortable answering any of the below questions you are welcome to skip any or all of those you do not wish to answer.

If applicable: What is the email address to which you wish to receive your transcript?

______________________________________________________________________________

**Demographics questions:**

1. **What is your age group?**

**O** <20 years

**O** 20-29 years

**O** 30-39 years

**O** 40-49 years

**O** 50-59 years

**O** ≥60 years

1. **What is your sex?**

**O** Male

**O** Female

**O** Prefer not to answer

1. **What is your current role?**

**O** Nurse

**O** Nurse practitioner

**O** Resident

**O** Fellow

**O** Attending physician

**O** Pharmacist

**O** Other (please specify): _________________

1. **[If applicable] What physician role do you identify as?**

**O** Primary clinician

**O** Clinician scientist

**O** Clinician educator

**O** Clinician administrator

**O** Other (please specify): ____________________

1. **How many years have you worked in your current role?**

**O** Please specify: ____________________

1. **How many years have you worked in critical care or within the hospital environment?**

**O** Please specify: ____________________

1. **What type of institution are you currently working in?**

**O** Academic

**O** Non-academic

**O** Regional

**O** Urban

**O** Other (please specify): ______________

1. **How many beds in total does your hospital have?**

**O** ≤250

**O** 251-499

**O** 500-1000

**O** >1000

**O** Other (please specify): ______________

1. **How big is the population your hospital serves?**

**O** Please specify: ____________________

1. **How many beds does your ICU or ward have?**

**O** Please specify: ____________________
